# Fertility Awareness and Attitudes Toward Parenthood Among University Students in Chitwan, Nepal: A Cross‐Sectional Study

**DOI:** 10.1002/puh2.70277

**Published:** 2026-05-15

**Authors:** Osin Gurung, Radha Devi Dhakal, Pushpa Sigdel, Anita Ghimire

**Affiliations:** ^1^ Department of Nursing Narayani Samudayik Hospital Bharatpur Nepal; ^2^ Shree College of Technology Affiliated to Purbanchal University Bharatpur Nepal

**Keywords:** attitude, fertility awareness, parenthood, university students

## Abstract

**Background:**

Fertility refers to the biological capacity to achieve pregnancy, whereas fertility awareness involves understanding the timing of the fertile window within the menstrual cycle. However, an increasing number of women and men are postponing parenthood, often without adequate awareness of age‐related fertility decline. This may lead to involuntary childlessness and infertility‐related stress. Fertility awareness among, particularly university students, remains limited, and evidence on their attitudes toward parenthood is insufficient. This study aimed to assess fertility awareness and attitudes toward parenthood among university students.

**Methods:**

A cross‐sectional analytical study was conducted among university students in Bharatpur, Chitwan, from March 15 to 30, 2024. A proportionate simple random sampling technique was used to select 415 respondents. Data were collected using a self‐administered structured questionnaire to assess fertility awareness and attitudes toward parenthood. The collected data were analyzed using Statistical Package for Social Sciences version 22 with descriptive statistical method (frequency, percentage) and inferential statistics (chi‐square, logistic regression).

**Results:**

The study found that more than half of the participants (54.4%) fall below the adequate level, with 30.8% exhibiting moderate level and 23.6% showing inadequate awareness and unfavorable attitudes toward parenthood (52.8%). Fertility awareness was significantly associated with marital status. Attitudes toward parenthood were significantly associated with age (adjusted odds ratios [AOR] = 3.54, 95% confidence intervals [CI]: 2.31–5.45, *p* < 0.001), ethnicity (AOR = 1.67, 95% CI: 1.07–2.60, *p* = 0.023), and religion (AOR = 1.90, 95% CI: 1.08–3.34, *p* = 0.025).

**Conclusion:**

Overall, university students demonstrated limited fertility awareness and less favorable attitudes toward parenthood. Knowledge gaps on fertility decline, ovulation, and infertility risk factors may lead to delayed decisions. Attitudes were significantly associated with age, ethnicity, and religion. These findings highlight the need for structured, age‐appropriate fertility education and counseling integrated into university curricula and services to support reproductive decision‐making.

## Graphical abstract

University students show limited fertility awareness and less favorable attitudes toward parenthood. Misconceptions about age‐related fertility decline and infertility risks may delay informed decisions. Attitudes vary by age, ethnicity, and religion, highlighting the need for structured, age‐appropriate fertility education and counseling to support informed reproductive choices.

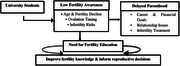

**Abbreviations**
AOR: adjusted odds ratio
CI: confidence interval
PHC: primary healthcare center
TFR: total fertility rate
UOR: unadjusted odds ratio
WHO: World Health Organization

## 1 Background of the Study

Fertility is defined as the capacity to establish a pregnancy [1]. Fertility awareness refers to knowledge of the timing of the fertile period and other biological and behavioral factors that affect an individual's ability to conceive [2]. It includes understanding reproductive processes, particularly the fertile window and factors influencing fertility, which support informed reproductive decision‐making [3]. However, inadequate fertility awareness can lead to delayed childbearing and involuntary childlessness [4], whereas infertility may negatively affect psychological and social well‐being [5].

Globally, fertility awareness has become an important public health concern, although most evidence originates from Europe and the United States [6, 7]. A large systematic review covering multiple countries reported substantial gaps in knowledge regarding age‐related fertility decline, the fertile window, and infertility risk factors [7]. At the same time, the postponement of parenthood has become a major global trend, driven by education, career aspirations, financial concerns, lifestyle choices, and delayed partnership formation [8, 9, 10]. Although such delays support personal and professional development, advancing age is associated with declining ovarian function and reduced oocyte quality [11]. Advanced maternal age is a key non‐modifiable risk factor for infertility [12] and is linked to adverse outcomes such as prematurity, fetal death, gestational complications, and increased cesarean delivery [13, 14]. Consequently, delayed childbearing is associated with higher risks of infertility, pregnancy complications, and increased reliance on assisted reproductive technologies (ART) [10].

In Nepal, fertility behavior has traditionally been influenced by strong sociocultural norms that highly value childbearing, with children considered essential for family continuity, social status, and economic and religious security [15]. Early marriage and expectations of childbearing soon after marriage remain common, often limiting women's autonomy in reproductive decision‐making [8]. However, fertility has declined substantially, with the total fertility rate (TFR) decreasing from 4.6 in 1996 to 2.1 in 2022 [9], largely due to improvements in education, urbanization, and access to family planning services [15]. The ideal family size has also shifted toward two children [16]. Reproductive health programs in Nepal have mainly focused on fertility reduction through family planning and contraception, with limited attention to fertility awareness [15]. However, recent community‐based interventions suggest that improving knowledge and addressing social norms can positively influence fertility‐related attitudes and decision‐making [17]. Evidence also indicates that both men and women in Nepal have inadequate awareness of fertility‐related issues [18].

As reproductive decisions are increasingly delayed, particularly among individuals pursuing higher education and career opportunities, understanding fertility awareness and attitudes toward parenthood has become essential. However, research in this area remains limited in Nepal. Therefore, this study was undertaken to assess fertility awareness and attitudes toward parenthood among university students.

## 2 Methods

### 2.1 Research Design Setting and Population

A cross‐sectional analytical study design was employed to assess fertility awareness and attitudes toward parenthood among university students. The study was conducted at Saptagandaki Multiple Campus, which was purposively selected due to its central location, high student enrollment, and representation of students from surrounding districts. These characteristics ensured the availability of an adequate and diverse sample population. The study population comprised male and female students currently enrolled in bachelor's degree programs across various faculties, including Bachelor of Science (BSc), Bachelor of Education (B.Ed.), Bachelor of Arts (BA), Bachelor of Business Studies (BBS), and Bachelor of Computer Application (BCA). Students who did not provide informed consent were excluded from the study.

### 2.2 Sample Size

The sample size was calculated using a prevalence of 44% for an adequate level of fertility awareness, on the basis of findings from a previous study [18]. Total population (*N*) = 844, sample size (*n*) = *Z* ^2^ *pq*/*e* ^2^ + (*Z* ^2^ *pq*/*N*). By using this formula, it is calculated as sample size (*n*) = (1.96)^2^ × 0.44 × 0.56/(0.05)^2^ + (1.96)^2^ × 0.44 × 0.56/844 = 378 + 0.0011 = 378, adding 10% non‐response rate = 378 + 37 = 415. So required sample size was 415.

### 2.3 Sampling Technique

A proportionate simple random sampling technique was used to select the study participants. The total study population consisted of 844 students from 5 faculties: BSc, B.Ed., BA, BBS, and BCA, with 104, 250, 210, 250, and 30 students respectively. From this population, a sample size of 415 was determined. To ensure proportional representation, a sampling fraction was calculated by dividing the total population by the required sample size (844/415 ≈ 2.03). On the basis of this proportion, the required number of participants from each faculty was 415.

After determining the required sample sizes, each student in the sampling frame was assigned a unique identification code. These codes were then placed in a closed container, and a lottery method was used to randomly select participants. The codes were drawn one by one until the required number of participants from each faculty was reached. Selected codes were kept separately to avoid duplication and were cross‐checked against the sampling frame. This process continued until the final sample size of 415 participants was obtained.

### 2.4 Data Collection Tool/Instrument

Data were collected using a semi structured, self‐administered questionnaire developed on the basis of an extensive literature review. The instrument comprised three sections: Part I assessed sociodemographic characteristics, Part II evaluated fertility awareness, and Part III measured attitudes toward parenthood using a Likert scale. Fertility awareness was assessed using 19 knowledge questions with response options of “Yes,” “No,” and “I don't know.” Each correct response was assigned 1 point, while incorrect and “I don't know” responses were scored as 0. Awareness levels were categorized on the basis of Bloom's standard cut‐off points: Scores of 80%–100% were considered high/adequate, 60%–79% as moderate, and below 60% as low/inadequate, with higher percentage indicating better fertility awareness [19]. Attitude toward parenthood were measured using a 5‐point Likert scale consisting of 14 items, with a total possible score of 70. Positive statements were scored from 5 (strongly agree) to 1 (strongly disagree), whereas negative statements were reverse scored from 1 (strongly agree) to 5 (strongly disagree). Scores above the mean indicated a favorable attitude, whereas scores equal to or below mean indicated an unfavorable attitude.

Content validity was ensured through consultation with the research advisor, research experts, and subject specialists. The questionnaire was translated from English into Nepali and subsequently back‐translated into English with the assistance of language experts to ensure linguistic accuracy. The reliability of the instrument was assessed through pretesting among 10% of the study population with similar inclusion criteria at a comparable institution (Birendra Multiple Campus). Items identified as ambiguous were revised for clarity, and language was simplified to ensure better understanding within the study context. Feedback from the pretest was used to refine the wording, making the questions more easily interpretable for the target population. Internal consistency was evaluated using Cronbach's alpha, which yielded a coefficient of 0.70, indicating acceptable reliability.

### 2.5 Ethical Considerations

Administrative approval was obtained from Shree College of Technology (SCOT), and ethical approval was secured from the Institutional Review Committee of Shree Medical and Technical College (SMTC‐IRC; Reference No. SMTC‐IRC‐20240303‐34). Permission was also obtained from the relevant university authorities prior to data collection. Written informed consent was obtained from all participants after clearly explaining the objectives and procedures of the study in understandable terms. Participants were informed of their right to refuse participation or withdraw from the study at any time without penalty. Confidentiality and anonymity were maintained by excluding personal identifiers and using the data solely for research purposes.

### 2.6 Data Collection Procedure

Formal permission to conduct the study was obtained from the concerned authorities, following submission of an official request letter from the college. Data were collected using a structured, self‐administered questionnaire distributed to male and female university students during break periods. The purpose and objectives of the study were clearly explained prior to questionnaire distribution, and informed consent was obtained from each participant. Participants were given approximately 25 min to complete the questionnaire, after which the completed forms were collected. Data collection was completed over a period of 2 weeks.

### 2.7 Data Analysis Procedure

All collected data were reviewed and organized on a daily basis to ensure completeness, consistency, and accuracy prior to entry. Data cleaning procedures were undertaken to identify and address missing values and outliers. The finalized dataset was coded and entered into Microsoft Excel and subsequently exported to SPSS version 22.0 for statistical analysis. Descriptive statistics, including frequencies, percentages, means, and standard deviations, were computed to summarize the data. The primary outcome variables of the study were the level of fertility awareness and attitude toward parenthood. Fertility awareness scores were categorized as adequate, moderate, and inadequate on the basis of Bloom's cutoff [19] criteria, whereas attitude scores were classified as favorable or unfavorable using the mean score as the threshold. The chi‐square test was employed to assess associations between sociodemographic variables and the outcome variables. Furthermore, logistic regression analysis was conducted to identify factors associated with attitudes toward parenthood. Variables found to be statistically significant in the bivariate analysis were included in the multivariate model to control for potential confounding effects. The strength of associations was presented using adjusted odds ratios (AOR) with 95% confidence intervals (CI). All statistical tests were two‐tailed, and a *p* value of less than 0.05 was considered statistically significant.

## 3 Results

Table 1 shows that the study participants were predominantly young, with 55.7% aged 15–20 years and a mean age of 20.74 ± 2.04 years. Females constituted 58.6% of respondents, and nearly half belonged to the Brahmin/Chhetri ethnic group (48.9%), whereas the majority practiced Hinduism (80.7%). Most respondents were unmarried (94.9%), unemployed (85.1%), and living with their family (80.2%), indicating limited current exposure to marital and parenting responsibilities. Academically, B.Ed. (29.6%) and BBS (29.4%) students formed the largest groups. Although still in young age, the majority of respondents (95.5%) reported having intentions regarding future childbearing. Regarding information sources, radio/TV/social media (57.1%) and the internet (52.3%) were the main sources, whereas only 9.6% received information from health workers or FCHVs.

**TABLE 1:** Sociodemographic information of the respondents (*n* = 415).

Variables	Frequency	Percent
**Age**		
15–20	231	55.7
20–25	184	44.3
Mean ± SD: 20.74 ± 2.039 Min(Max) 17(24)		
**Sex**		
Male	172	41.4
Female	243	58.6
**Ethnicity of respondent**		
Dalit	74	17.8
Janajati	110	26.5
Madhesi	21	5.1
Muslim	7	1.7
Brahmin/Chhetri	203	48.9
**Religion of respondent**		
Hinduism	335	80.7
Buddhism	66	15.9
Islam	6	1.4
Christianity	8	1.9
**Employment status of respondent**		
Employed	62	14.9
Unemployed	353	85.1
**Academic status of respondent**		
BBS	122	29.4
B.Ed.	123	29.6
BA	103	24.8
BSc	52	12.5
BCA	15	3.6
**With whom respondent live**		
Alone	48	11.6
Family	333	80.2
Same gender friend	34	8.2
**Marital status**		
Unmarried	394	94.9
Married	21	5.1
**Plan to have child in future**		
Yes	396	95.4
No	19	4.6
**Source of information**		
Family member	75	18.1
Friends	124	29.9
Radio/TV/social media	237	57.1
Internet	217	52.3
FCHV/health worker	40	9.6

Abbreviations: B.Ed., Bachelor of Education; BA, Bachelor of Arts; BBS, Bachelor of Business Studies; BCA, Bachelor of Computer Application; BSc, Bachelor of Science.

Table 2 presents moderate but incomplete fertility knowledge among university students. Although 72.8% correctly understood the meaning of fertility and 73.7% accurately identified the fertile period for conception, knowledge gaps were evident in core biological concepts. Only 52.2% correctly defined ovulation, and less than half correctly identified the timing of ovulation (49.8%) and the age at which sperm production begins (48.9%). Awareness of age‐related fertility decline was also limited, with 57.8% correctly identifying the marked decline in female fertility at 35–39 years and 61.4% recognizing decreased male fertility after 40 years. Knowledge of infertility was relatively low, as only 47.0% correctly defined infertility as failure to conceive after 1 year of unprotected intercourse. Although the awareness of major infertility risk factors responded to hormonal imbalance (78.8%), fewer respondents recognized reproductive tract infections (44.6%) as risk factors. Understanding of common causes of infertility was comparatively better, with 70.6% correctly identifying low sperm count as the most common cause of male infertility and 62.8% identifying lack of ovulation as a leading cause of female infertility.

**TABLE 2:** Knowledge of respondents regarding fertility awareness (*n* = 415).

Questions	Correct answer	Frequency	Percent
Meaning of fertility	Ability to conceive and reproduce children	302	72.8
Most fertile age of women	20–24	278	67.0
Marked decrease in women fertility	35–39	240	57.8
Most fertile age of men	26–29	282	68.0
Marked decrease in male fertility	>40	255	61.4
Meaning of ovulation	Release of egg from ovaries	217	52.2
Time period of ovulation	14 days before the start of next menstrual period	207	49.8
Time period of conceive	14 days before the start of next menstrual period	306	73.7
Age of start of sperm production	12–13	203	48.9
True statement about infertility	More than 1 year of regular unprotected intercourse	195	47.0
Risk factor for infertility	Hormonal imbalance	327	78.8
	Smoking	256	61.7
	Exposure to chemical and radiation	324	78.1
	Alcohol consumption	270	65.1
	Elderly age	287	69.2
	Obesity	193	46.5
	Reproductive tract infection	185	44.6
Common cause of male infertility	Low sperm count	293	70.6
Common cause of female infertility	Lack of ovulation	261	62.8

Table 3 demonstrates the attitude findings indicate a mixed but generally less positive perception of parenthood among students. The highest proportion of respondents (48.4%) strongly agreed that parenthood makes a person feel complete as a woman or man, and nearly half (49.6%) strongly agreed that parenthood helps establish a stronger relationship between partners, reflecting positive emotional and relational perceptions. A clear majority also agreed that relationship and support systems (45.5%) and personal values and beliefs (55.2%) play an important role in shaping parenthood decisions. Regarding challenges, the largest proportion agreed that emotional stress affects the ability to navigate parenthood confidently (58.3%) and that delaying marriage leads to delayed parenthood (58.3%). Financial concerns were prominent, with 45.5% strongly agreeing that parenthood increases expenses. In contrast, the highest responses for several negative perceptions were disagreement, with 31.1% disagreeing that parenthood leads to loss of freedom, 37.3% strongly disagreeing that being a parent makes a person tense and anxious, and 40.5% strongly disagreeing that parenthood strains partner relationships. Additionally, most respondents (57.6%) strongly disagreed that parenthood varies according to the gender of children, indicating low endorsement of gender‐based child preferences.

**TABLE 3:** Attitude of respondents towards parenthood (*n* = 415).

Statement	SA	A	N	D	SD
Phase parenthood make person feel complete as woman/man	201 (48.4)	105 (25.3)	35 (8.4)	64 (15.4)	10 (2.4)
Person who belongs to broken family doesn't prefer parenthood	14 (3.4)	70 (19.9)	96 (23.1)	183 (44.1)	52 (12.5)
Family relationship and support system affect perception of being parent	148 (35.7)	189 (45.5)	66 (15.9)	5 (1.2)	7 (1.7)
Personal values and beliefs on family play a role towards starting family	130 (31.3)	229 (55.2)	42 (10.1)	8 (1.9)	6 (1.4)
Emotional stress affect ability to navigate parenthood confidently	91 (21.9)	242 (58.3)	51 (12.3)	14 (3.4)	1 (0.2)
Delay marriage leads to delay parenthood	91 (21.9)	242 (58.3)	51 (12.3)	20 (4.8)	11 (2.7)
Parenthood leads to loss of freedom	38 (9.2)	96 (23.1)	85 (20.5)	129 (31.1)	67 (16.1)
Parenthood helps to establish stronger relationship between partner	206 (49.6)	134 (32.3)	55 (13.3)	14 (3.4)	6 (1.4)
There is less time to devote to work and career after having child	34 (8.2)	89 (21.4)	135 (32.5)	121 (29.2)	36 (8.7)
Being parent makes tense and anxious	19 (4.6)	66 (15.9)	73 (17.6)	102 (24.6)	155 (37.3)
Parenthood leads to increase in expenses	189 (45.5)	152 (36.6)	42 (10.1)	19 (4.6)	13 (3.1)
After having child there is less time for own interests	32 (7.7)	82 (19.8)	106 (25.5)	160 (38.6)	35 (8.4)
Parenthood leads to strain on relationship between partner	19 (4.6)	63 (15.2)	65 (15.7)	100 (24.1)	168 (40.5)
Parenthood can vary according to gender of children	20 (4.8)	71 (17.1)	38 (9.2)	47 (11.3)	239 (57.6)

Abbreviations: A, agree; D, disagree; N, neutral; SA, strongly agree; SD, strongly disagree.

The findings of Table 4 show that the distribution of awareness levels among participants indicates a mixed pattern of knowledge. Nearly half of the respondents (45.5%) demonstrated adequate awareness, suggesting that a considerable proportion possesses satisfactory understanding. However, more than half of the participants (54.4%) fall below the adequate level, with 30.8% exhibiting moderate awareness and 23.6% showing inadequate awareness. The mean awareness score was (12.59), reflecting moderate but insufficient understanding of fertility‐related concepts. In terms of attitudes, a slightly higher proportion of students (52.8%) exhibited an unfavorable attitude toward parenthood, whereas 47.2% had a favorable attitude. The mean attitude score (51.74) suggests mixed perceptions, with a tendency toward less favorable attitudes.

**TABLE 4:** Level of fertility awareness and attitude towards parenthood (*n* = 415).

Level of awareness	Frequency	Percent
Adequate (80%–100%)	189	45.5
Moderate (60%–79%)	128	30.8
Inadequate (<60%)	98	23.6
Mean ± SD: 12.59 ± 2.43 (min 6, max 17)		
**Level of attitude**		
Favorable	196	47.2
Unfavorable	219	52.8
Mean ± SD: 51.74 ± 9.068 (min 28, max 67)		

Table 5 reveals that age, ethnicity, and religion were significantly associated with students’ attitudes. Younger students aged 15–20 years were more likely to have an unfavorable attitude toward parenthood compared to those aged 20–25 years (AOR = 3.54, 95% CI: 2.31–5.45, *p* < 0.001), indicating that older students may hold more favorable perspectives. Students from the Brahmin/Chhetri ethnic group had higher odds of a favorable attitude compared to other ethnicities (AOR = 1.67, 95% CI: 1.07–2.60, *p* = 0.023), and Hindu students were more likely to have a favorable attitude than students from other religions (AOR = 1.90, 95% CI: 1.08–3.34, *p* = 0.025). Although employment status and living arrangement were significant in unadjusted analysis, these associations were not maintained after adjustment. Sex, marital status, and childbearing plan were not significantly associated with the level of attitude in both unadjusted and adjusted analyses.

**TABLE 5:** Association between level of attitude toward parenthood and sociodemographic variables.

Variables		Level of attitude					
	Category	Unfavorable	Favorable	UOR (95% CI)	*p* value	AOR (95% CI)	*p* value
Age in year	15–20	140 (60.60)	91 (39.40)	3.516 (2.333–5.300)	0.043	3.544 (2.306–5.447)	0.001
	20–25	56 (30.40)	128 (69.60)	Ref		Ref	
Sex	Male	78 (45.30)	94 (54.70)	1.138 (0.769–1.683)	0.519	1.12 (0.732–1.713)	0.602
	Female	118 (48.60)	125 (51.40)	Ref		Ref	
Ethnicity	Brahmin/Chhetri	80 (39.40)	123 (60.60)	1.858 (1.257–2.745)	0.002	1.67 (1.073–2.601)	0.023
	Other than Brahmin/Chettri	116 (54.70)	96 (45.30)	Ref		Ref	
Religion	Hinduism	147 (43.90)	188 (56.10)	2.022 (1.227–3.329)	0.006	1.902 (1.083–3.340)	0.025
	Other than Hinduism	49 (61.30)	31 (38.80)	Ref		Ref	
Employment status	Employed	24 (35.80)	43 (64.20)	1.751 (1.019–3.01)	0.043	1.386 (0.753–2.552)	0.295
	Unemployed	172 (49.40)	176 (50.60)	Ref		Ref	
Live with	Family	144 (44.40)	180 (55.60)	1.667 (1.042–2.665)	0.033	1.322 (0.78–2.239)	0.299
	Others	52 (57.10)	39 (42.90)	Ref		Ref	
Marital status	Unmarried	187 (47.90)	207 (52.50)	1.205 (0.496–2.923)	0.681	1.234 (0.436–3.496)	0.295
	Married	9 (42.90)	12 (57.10)	Ref		Ref	
Child plan	Yes	7 (41.20)	10 (58.80)	1.292 (0.482–3.462)	0.611	1.165 (0.146–9.275)	0.885
	No	189 (47.50)	209 (52.50)	Ref		Ref	

*Note:* Significance at 0.05 level, 1 = reference group, significant at 95% CI.

Abbreviations: AOR, adjusted odds ratio; UOR, unadjusted odds ratio.

Concerning association between level of fertility awareness and sociodemographic variables, the chi‐square test indicated that only marital status (*p* = 0.049) was significantly associated with fertility awareness, whereas all other variables were not significantly associated.

## 4 Discussion

This study examined fertility awareness and attitudes toward parenthood among university students. The findings indicate a substantial lack of fertility awareness, particularly regarding the biological limits of reproduction and age‐related fertility decline. Similar deficiencies in fertility knowledge have been reported among university students and young adults in Sweden, the United States, and Hong Kong, suggesting that low fertility awareness is a global concern rather than a context‐specific issue [10, 20, 21, 22]. Limited understanding of fertility may influence reproductive decision‐making and contribute to delayed childbearing observed worldwide [9, 23].

Globally, declining TFRs have been associated with delayed marriage, postponed parenthood, and changing life priorities among young adults [24]. In the present study, most respondents without children expressed willingness to become parents in the future and reported a desired family size of two children. This finding aligns with demographic patterns indicating that fertility intentions remain positive despite delayed timing. In Nepal, the TFR has declined markedly from 4.6 in 1996 to 2.1 in 2022, reflecting rapid social and demographic transitions influenced by education, urbanization, and women's workforce participation [9].

Previous studies that reported lower fertility awareness among male participants, the present study found no significant gender differences in overall fertility knowledge [9]. This finding is consistent with evidence from Denmark, where comparable levels of fertility awareness were observed among male and female students. Regarding academic discipline, the present study found no significant association between field of study and fertility awareness. This contrasts with studies from Australia and the United Kingdom, which have highlighted higher fertility knowledge among students enrolled in health‐related disciplines compared to those from non‐health science backgrounds [7, 25]. However, even in those studies, notable knowledge gaps persisted, suggesting that general reproductive health education at the tertiary level may be insufficient to comprehensively address fertility‐related issues.

In this study, although many respondents believed that they knew the most fertile age range for men and women, a considerable proportion lacked accurate knowledge about when fertility begins to decline significantly. Similar inconsistencies have been reported in other studies, where young adults tend to overestimate both the age of female fertility decline and the success rates of ART [22, 26]. Such overestimations may lead to unrealistic expectations regarding future reproductive potential.

In terms of infertility risk factors, students showed the awareness of smoking, tobacco use, substance abuse, hormonal imbalance, and lifestyle‐related factors. However, more than half of the respondents did not recognize obesity, infections, or sexually transmitted infections (STIs) as risk factors for infertility, despite strong evidence linking these conditions to impaired reproductive health outcomes [27, 28]. Similar misconceptions have been reported in European and North American studies, where young adults often associate fertility decline primarily with menopause rather than gradual age‐related changes across the reproductive lifespan [20, 29]. Unhealthy lifestyle behaviors, including high‐fat diets, excessive caffeine consumption, physical inactivity, occupational stress, and increased scrotal temperature in men, have also been shown to negatively affect fertility [10, 23].

With regard to attitudes toward parenthood, more than half of the respondents demonstrated a negative overall attitude, although the majority still perceived parenthood as an important life stage contributing to a sense of personal completeness. Attitudes toward balancing work and career after having children were mixed, with a substantial proportion remaining neutral. Students generally expressed positive intentions toward future parenthood but preferred to delay childbearing until educational and career goals were achieved. This pattern reflects global trends among highly educated youth, where parenthood is viewed as important but not urgent [8, 18, 30]. Financial insecurity, employment instability, and gendered expectations related to household and childcare responsibilities have been identified as key barriers to earlier childbearing. Study [31] reported that financial burden combined with women's disproportionate responsibility for household and childcare duties negatively influences fertility intentions. Evidence from South Korea further indicates that increasing job competition and labor market insecurity among young adults interfere with partnership formation and childbearing plans [24]. Similar structural and economic pressures may be shaping fertility attitudes and behaviors among Nepalese university students.

Regarding sources of fertility information, most students reported relying on radio, television, and social media, whereas healthcare professionals were the least cited source. This finding highlights a critical gap in the role of healthcare providers in fertility education. Previous studies emphasize that physicians and other healthcare workers may themselves have limited training in fertility counseling, which restricts their engagement in preventive fertility education [3, 21]. Evidence suggests that male fertility is influenced by advancing age, unhealthy lifestyle behaviors, and environmental exposures [10]. Although female fertility is also affected by increasing age and related biological factors [32] underscoring the need for accurate professional guidance.

This study was conducted in a single setting, which may limit the generalizability of the findings. Data were collected using a self‐administered questionnaire due to time and budget constraints. The study population was limited to university students, which may not represent the general population. Additionally, the use of a newly developed instrument may limit comparability with other studies, and dichotomization of Likert scale responses may cause loss of information and potential bias by oversimplifying participants’ attitudes. Future research with larger, more diverse samples, and face‐to‐face data collection method is recommended to validate and expand these findings.

## 5 Conclusions

Overall, the study shows that university students have limited fertility awareness and less favorable attitudes toward parenthood. Many lack accurate knowledge of age‐related fertility decline, ovulation timing, sperm production, and infertility risk factors, which may lead to delayed and uninformed decisions. Although parenthood is perceived as meaningful and beneficial for relationships, concerns about emotional stress, financial burden, and life timing may discourage early parenthood. These findings highlight the need for structured, age‐appropriate fertility education and counseling within university settings to support informed reproductive decision‐making.

## Author Contributions

**Osin Gurung**: conceptualization, investigation, funding acquisition, methodology, writing – review and editing, visualization, validation, data curation, resources, software, project administration, formal analysis, writing – original draft. **Anita Ghimire**: supervision, resources, project administration, writing – review and editing, writing – original draft. **Pushpa Sigdel**: data curation, writing – review and editing, writing – original draft, formal analysis. **Radha Devi Dhakal**: conceptualization, methodology, software, data curation, supervision, resources, visualization, validation, writing – review and editing, writing – original draft, formal analysis.

## Funding

The authors have nothing to report.

## Disclosure

This study was conducted as part of the corresponding author (Radha Devi Dhakal) undergraduate Nursing degree requirements at the Purbanchal University, of Shree College of Technology.

## Ethics Statement

Ethical approval was obtained from SMTC‐IRC‐20240303‐34; formal permission was obtained from the concerned authorities for data collection.

## Consent

Written informed consent was obtained prior to data collection.

## Conflicts of Interest

The authors declare no conflicts of interest.

## Data Availability

The data that support the findings of this study are available from the corresponding author on reasonable request because the scope of the data and consent obtained from study participants restrict our ability to share the data on ethical and legal rules.
